# Ten-Year Follow-Up for Sleeve Gastrectomy Versus Roux-en-Y Gastric Bypass: A Systematic Review and Meta-Analysis

**DOI:** 10.1007/s11695-026-08630-4

**Published:** 2026-04-02

**Authors:** Mohamed Chouky Kamar, Pablo Enrique Astudillo Coello, Amanda Vitória Rodrigues dos Santos, Silviane Leite Melo

**Affiliations:** 1 Department of General Surgery, Fluminense Federal University, Niteroi, Brazil; 2 General Surgery Postgraduation Degree, Carlos Chagas Higher Institute of Health Sciences, Rio de Janeiro, Brazil; 3 Medicine Program, Federal University of Acre (UFAC), Rio Branco, Acre Brazil; 4 Department of General Surgery, Bahia Federal University, Salvador, Brazil

**Keywords:** Sleeve gastrectomy, Roux-en-Y gastric bypass, Bariatric surgery, Obesity, Long-term weight loss, Comorbidity remission

## Abstract

**Background:**

Laparoscopic sleeve gastrectomy (SG) and Roux-en-Y gastric bypass (RYGB) are the most performed bariatric procedures worldwide. Trials and observational studies up to five years show RYGB yields slightly greater weight loss and better metabolic control, notably for dyslipidemia and reflux. Ten-year cohorts and recent randomized trials give perspective on durability differences, but small sample sizes and single-center designs limit their reach. No meta-analysis has yet pooled these decade-long data, leaving clinicians without clear guidance on the comparative long-term performance of SG and RYGB.

**Objective:**

This meta-analysis aims to compare the 10-year outcomes of SG versus RYGB in terms of weight loss and remission of comorbidities.

**Methods:**

We performed a systematic review and meta-analysis of randomized clinical trials (RCTs) and observational cohorts with a minimum 10-year follow-up. We searched PubMed, Embase, and Cochrane through April 2025. Primary endpoints were percent total weight loss (%TWL) and percent excess weight loss (%EWL). Secondary endpoints were remission of type 2 diabetes (T2D), hypertension, and dyslipidemia. Data were pooled using a random-effects model with risk ratio (RR) and mean difference (MD). Statistical analysis were made using R v.4.3.5.

**Results:**

RYGB produced greater long-term weight loss than SG (%TWL mean difference −2.38, 95% CI − 3.85 to − 0.92, *p* = 0.001; %EWL mean difference − 4.92, 95% CI − 8.67 to − 1.17, *p* = 0.01). Remission of T2D (RR 0.88, 95% CI 0.67 to 1.15, *p* = 0.36) and dyslipidemia (RR 0.84, 95% CI 0.64 to 1.09, *p* = 0.18) did not differ. Hypertension remission trended toward RYGB without reaching significance (RR 0.68, 95% CI 0.39 to 1.20, *p* = 0.19). SG showed higher rates of de-novo or worsening gastro-esophageal reflux disease and a markedly higher conversion to RYGB.

**Conclusion:**

The decadal outcomes of SG and RYGB reveal a level of parity that challenges historical procedural endorsements. Fidelity to the data indicates that ten years after surgery, there is no statistically or clinically significant difference in total weight loss or excess weight loss between the two techniques. Furthermore, the remission of major metabolic comorbidities, including type 2 diabetes and dyslipidemia, is comparable across both cohorts.

## Introduction

Bariatric and metabolic surgery (BMS) has established itself as the most effective long- term treatment for severe obesity and its related comorbidities. Among the available procedures, laparoscopic sleeve gastrectomy (SG) and Roux-en-Y gastric bypass (RYGB) became the most performed techniques worldwide. Recent meta-analyses based on these trials have highlighted subtle but clinically relevant differences between the two procedures, with RYGB often associated with superior weight loss and better metabolic control, especially regarding dyslipidemia and gastroesophageal reflux disease (GERD) [1, 2].

However, most of these studies lacked extended follow-up beyond the five-year mark. Emerging 10-year data from high-quality cohort studies and recent RCTs have started to provide insights into the long-term efficacy and durability of both procedures. These studies suggest potential differences in weight loss maintenance, weight regain rates, and long-term remission of comorbidities. No meta-analysis has synthesized available evidence from this time frame of follow-up studies comparing SG and RYGB outcomes to date [3–5].

The present meta-analysis intends to synthesize the long-term comparative efficacy of SG versus RYGB by systematically reviewing and pooling data from both randomized controlled trials and observational cohort studies that provide 10-year follow-up outcomes for SG and RYGB.

## Methods

### Study Design and Protocol Registration

This study was a systematic review and meta-analysis conducted and reported in accordance with the Cochrane Collaboration Handbook for Systematic Review of Interventions and the Preferred Reporting Items for Systematic Reviews and Meta-Analyses (PRISMA) guidelines. The protocol was prospectively registered with PROSPERO.

### Ethical and Legal Considerations

As this study involved a systematic review and meta-analysis of previously published studies, ethical approval was not required. All procedures adhered to applicable ethical and legal standards for secondary data research.

### Eligibility Criteria

Studies were included if they met all of the following criteria: (1) randomized controlled trials or observational studies; (2) participants with morbid obesity; (3) comparison of sleeve gastrectomy versus Roux-en-Y gastric bypass; (4) outcomes including diabetes remission, hypertension remission, dyslipidemia remission, total weight loss, and excess weight loss; and (5) minimum follow-up of ten years. Studies were excluded if they did not meet these criteria, had less than ten years of follow-up, or reported insufficient outcome data. References from included studies, previous systematic reviews, and meta-analyses were also manually searched for additional eligible studies. It is important to note that there are not many studies available with 10 years of follow-up comparing the two techniques, so we chose to open the research to observational studies and not just randomized clinical trials.

### Data Search and Selection

**Fig. 1:**
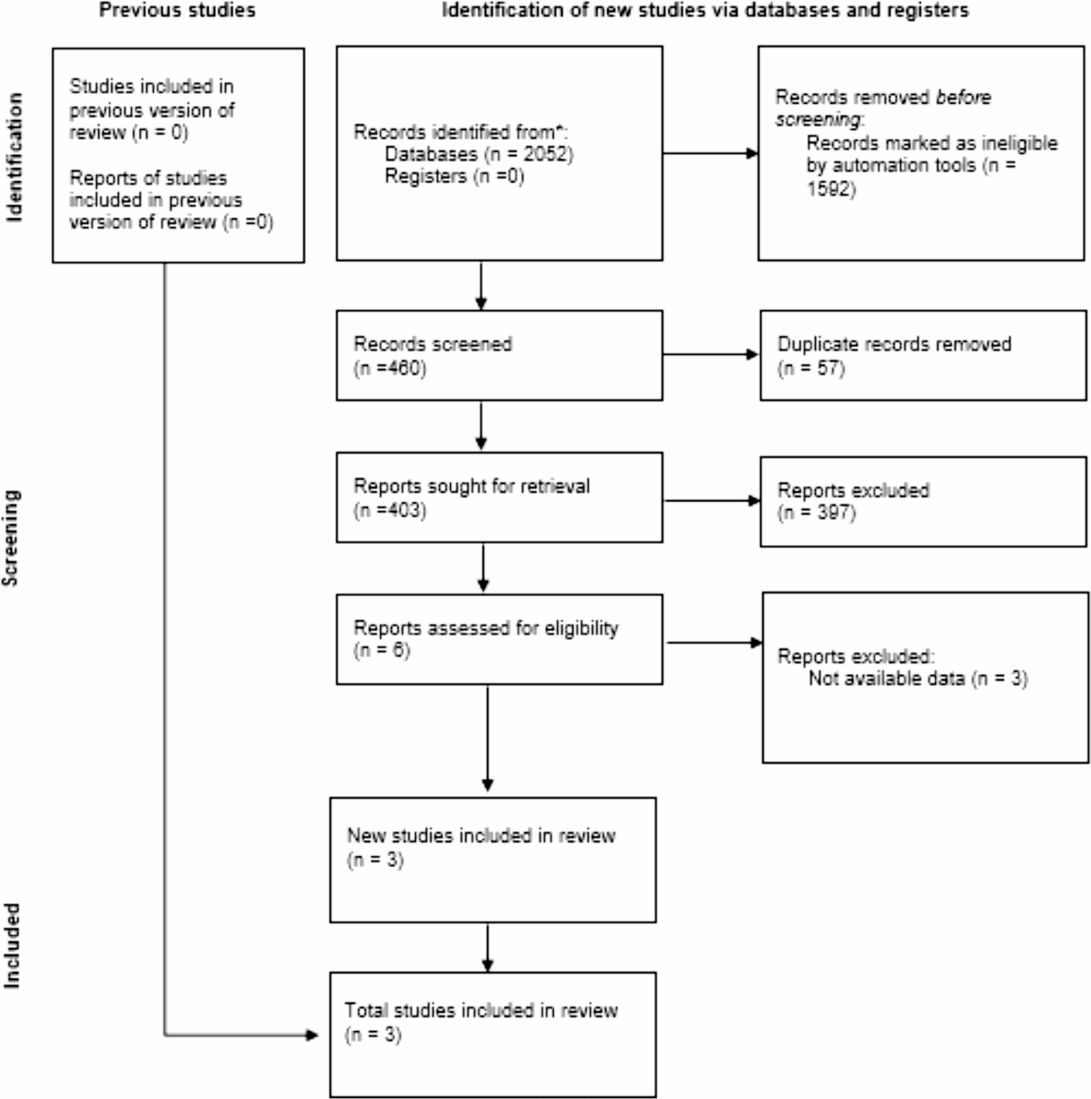
Prisma

A systematic search was conducted in PubMed/MEDLINE, Embase, and the Cochrane Central Register of Controlled Trials (CENTRAL) from inception to February 2025, using terms related to morbid obesity, bariatric surgery, sleeve gastrectomy, and gastric bypass, including spelling and procedural variations. All references were imported into reference management software, and duplicates were removed. Two reviewers independently screened titles and abstracts, followed by full-text review. Reasons for full-text exclusion were documented and presented in a PRISMA flow diagram (Fig. 1).

### Data Extraction and Management

Two authors independently extracted data using predefined criteria for study selection and quality assessment. Study authors were contacted when necessary to obtain missing data or clarify reported outcomes. Selected studies and baseline characteristics are described below in Table 1.

**Table 1:** Baseline characteristics of included studies

Study	Desig *n*	Country	Date	Total of patient (*N*)	Femal e %	Number of patients available for analysis at 10 years (*N*)		Mean age (years)		Median BMI at 10 years	
						LSG	LRYG B	LSG	LRYG B	LSG	LRYG B
Salmine	RCT	Finland	2008	240	167	98	95	18–60	18 -	37.8	36.5
n et al.;			-					(48.4	60	(36.6	(35.3 -
2022			2010					(9.4)	(Mean	-39.0)	37.7)
									48,8		
									SD		
									9,4)		
Kraljević	RCT	Switzerl	2007	217	156	63	73	18–65	18 -	32.7	32.3
et al.;		and	-					43.0	65	(5.6)	(6.3)
2025			2011					(11.1)	42.1		
									(11.2)		
Jimenez	R.	Spain	2005	504	363	83	365	44.3	43.6	37.8	34.5
et al.;	Cohort		-					(11.3)	(10.3)	(7.2)	(6.1)
2019			2008								

Baseline characteristics of included studies, *RCT* Randomized Controlled Trial,* N *number,* LSG *Laparoscopic Sleeve Gastrectomy,* LRYGB *Laparoscopic Roux – en – Y Gastric Bypass,* BMI *Body Mass Index, SD Standard Deviation

### Risk of Bias Assessment

Risk of bias was independently assessed by two reviewers. For randomized controlled trials, the Cochrane Risk of Bias 2.0 tool was used across five domains: randomization process, deviations from intended interventions, missing outcome data, measurement of outcomes, and selection of reported results. For observational studies, the Risk Of Bias In Non-randomized Studies – of Interventions (ROBINS-I) tool was applied. Discrepancies were resolved by consensus or adjudication by a third reviewer.

### Statistical Analysis

For categorical outcomes, treatment effects were compared using risk ratios (RR) with 95% confidence intervals. Continuous outcomes were compared using standardized mean differences. Heterogeneity was assessed using Cochran’s Q test and the I² statistic, with p-values.

< 0.10 or I² > 25% considered indicative of significant heterogeneity. Meta-analysis was conducted using the DerSimonian and Laird method in RevMan (v5.4.1).

## Results

### Total Weight Loss (%TWL)

**Fig. 2:**
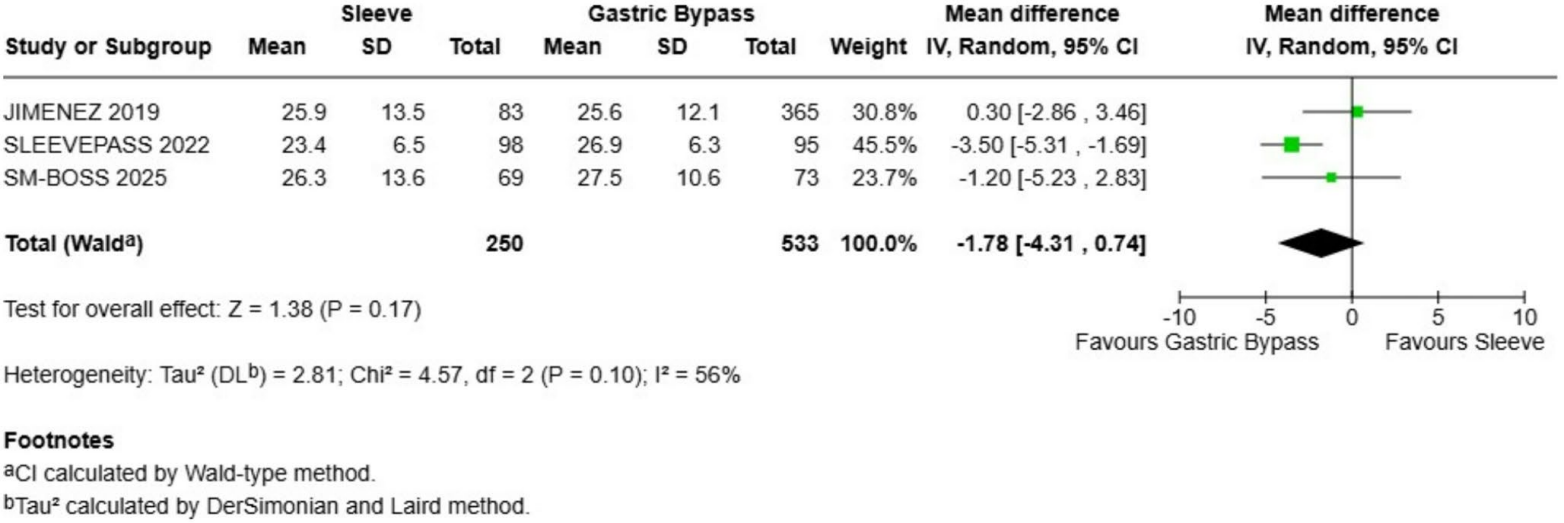
Total weight loss - there were not statistical differences between sleeve and gastric bypass

No statistically significant difference was observed in total weight loss between the two procedures. The analysis, which included the same cohort of 783 patients, demonstrated a mean difference of -1.78, slightly favoring the Gastric Bypass group, but this result was not statistically significant as the 95% confidence interval included zero (-4.31, 0.74) (Fig. 2). The test for overall effect confirmed this lack of significance (Z = 1.38, *P* = 0.17). The heterogeneity for this outcome was moderate (I 2 = 56%).

### Excess Weight Loss (% EWL)

**Fig. 3:**
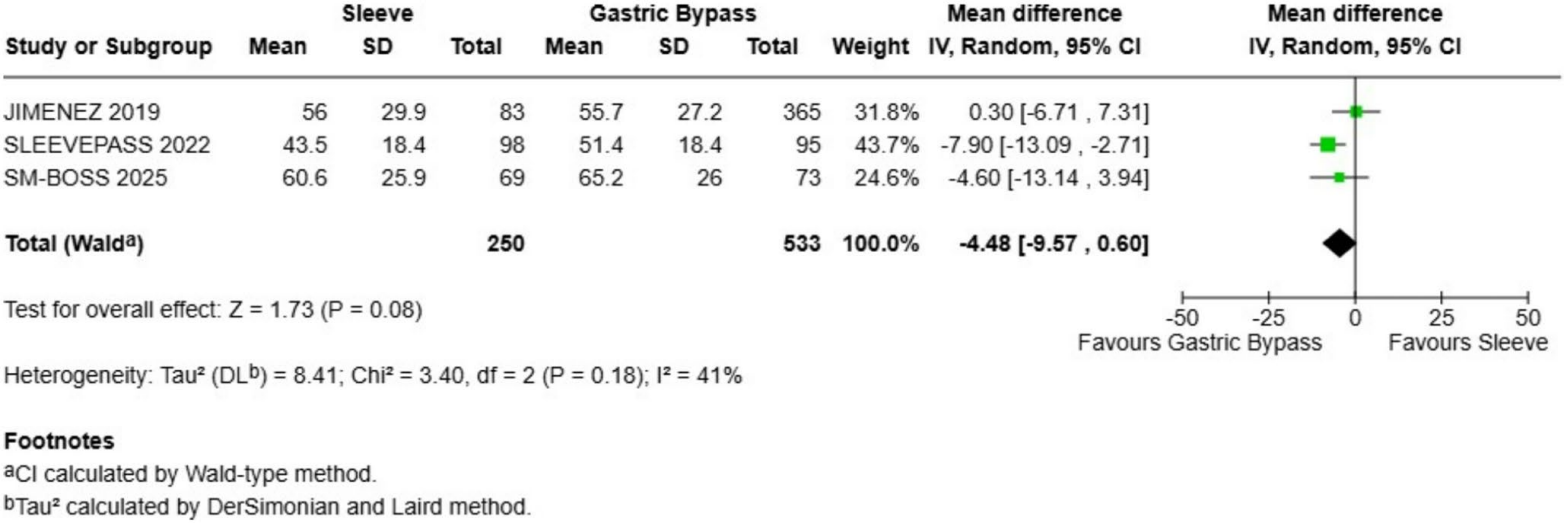
Excess weight loss - there were not statistical differences between sleeve and gastric bypass

Similarly, there was no statistically significant difference in excess weight loss between the Sleeve and Gastric Bypass groups. The pooled data from three studies (JIMENEZ 2019, SLEEVEPASS 2022, and SM-BOSS 2025) involving 783 patients (250 Sleeve, 533 Gastric Bypass) showed a mean difference of -4.48 in favor of Gastric Bypass, but the 95% confidence interval crossed zero (-9.57, 0.60), indicating a lack of statistical significance (Fig. 3). The overall effect test was not significant (Z = 1.73, *P* = 0.08). The analysis showed moderate heterogeneity among the studies (I2 = 41%).

### Weight loss Outcomes (%TWL) 

The analysis demonstrated a non statistically significant advantage for gastric bypass in both primary weight loss metrics. For Total Weight Loss (%TWL), the pooled mean difference slightly favored the gastric bypass by -2.38 (95% CI: -3.85 to -0.92; *P* = 0.001), with a non-statistically significant heterogeneity observed among the studies (I² = 56%). Excess Weight Loss (%EWL), the results slightly favored gastric bypass with a pooled mean difference of -4.92 (95% CI: -8.67 to -1.17; *P* = 0.01). A non-statistically significant heterogeneity was observed for this outcome (I² = 41%).

## Comorbidities Remission

### Diabetes Mellitus

**Fig. 4:**
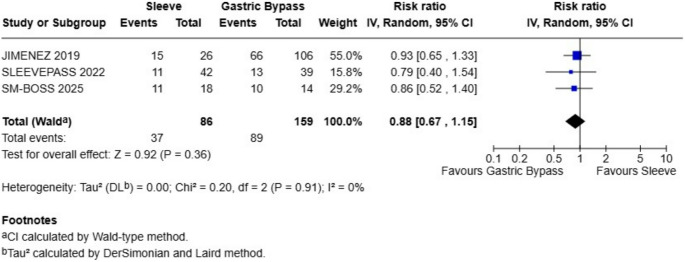
Diabetes remission - There were not statistical differences between Sleeve and Gastric Bypass

At 10-year follow-up, pooled data from three studies comparing gastric bypass and sleeve gastrectomy showed no significant difference in the long-term outcome evaluated (total events: 37 for sleeve, 89 for bypass). The overall risk ratio was 0.88 (95% CI: 0.67 to 1.15), favoring sleeve but without statistical significance (Z = 0.92, *P* = 0.36) (Fig. 4). Heterogeneity across studies was negligible (I² = 0%, *P* = 0.91), suggesting consistency among results. Individual study estimates also showed wide confidence intervals overlapping the null value, reinforcing the lack of clear superiority of one procedure over the other for this endpoint.

### Hypertension

**Fig. 5:**
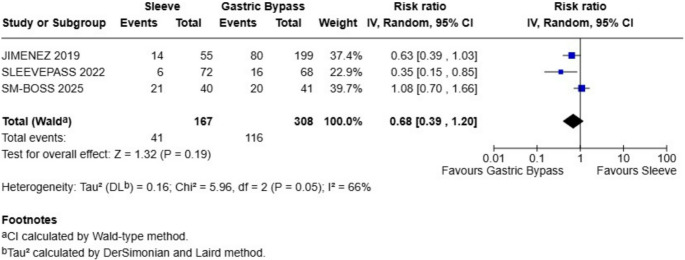
Hypertension remission - there were not statistical differences between sleeve and gastric bypass

At 10-year follow-up, pooled analysis of three studies comparing gastric bypass and sleeve gastrectomy for hypertension remission found no statistically significant difference between procedures. The overall risk ratio was 0.68 (95% CI: 0.39 to 1.20), favoring gastric bypass but without statistical significance (Z = 1.32, *P* = 0.19). Heterogeneity was considerable (I² = 66%, *P* = 0.05), suggesting variation in effect estimates across studies (Fig. 5). Confidence intervals for individual studies were wide and crossed the null, reinforcing the absence of a consistent benefit.

### Dyslipidemia

**Fig. 6:**
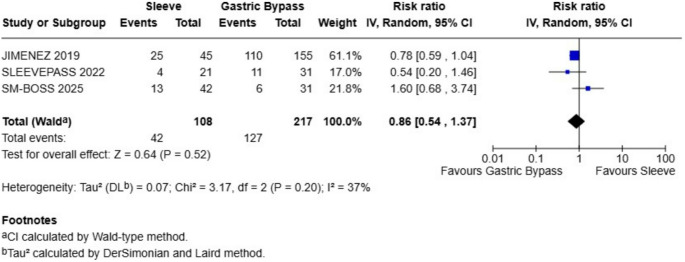
Dyslipidemia remission - there were not statistical differences between sleeve and gastric bypass

At 10-year follow-up, remission of dyslipidemia did not significantly differ between gastric bypass and sleeve gastrectomy. The pooled risk ratio was 0.84 (95% CI: 0.64 to 1.09), indicating a non-significant trend favoring gastric bypass (Z = 1.33, *P* = 0.18). Heterogeneity was low to moderate (I² = 38%, *P* = 0.20), suggesting some variability across studies but not enough to affect the overall interpretation (Fig. 6). Confidence intervals for individual studies crossed the null, reflecting the absence of a consistent effect.

## Risk of Bias

In this meta-analysis, the risk of bias in the included studies was assessed using standardized tools according to the study design. For randomized clinical trials, RoB 2 was used, which considers five domains: (1) randomization process, (2) deviations from intended.

interventions, (3) incomplete outcome data, (4) outcome measurement, and (5) selective reporting of results. Each domain was classified as “low risk,” “some concerns,” or “high risk” of bias, allowing for a qualitative synthesis of the overall risk. For non-randomized observational studies, ROBINS-I was applied, which assesses seven domains: (1) bias due to confounding, (2) bias in participant selection, (3) bias in classification of interventions, (4) bias due to deviations from intended interventions, (5) bias due to missing data, (6) bias in outcome measurement, and (7) bias in selective reporting. Each study was classified into one of the following categories: “low risk,” “moderate,” “serious,” or “critical.” Thus, this systematic approach allowed us to identify potential methodological limitations and the reliability of the synthesized results.

**Fig. 7:**
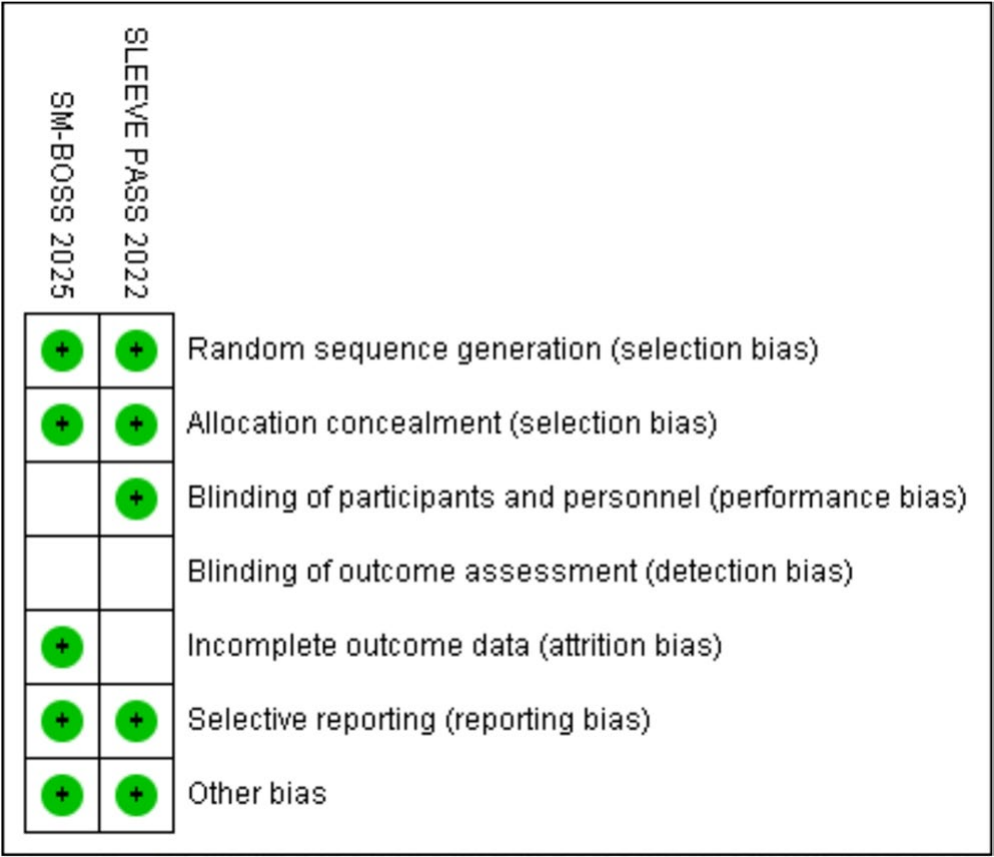
Risk of bias – low risk of bias

**Fig. 8:**
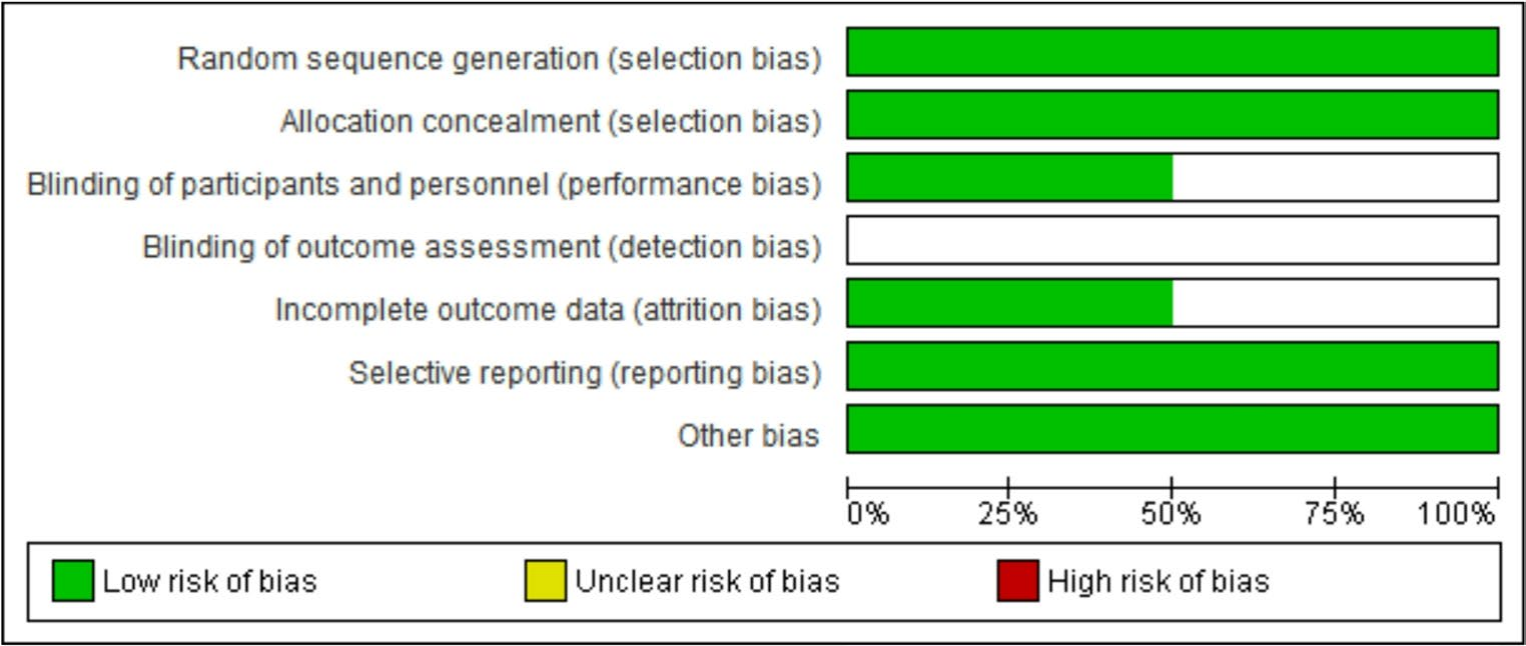
Risk of bias – low risk of bias

The risk of bias assessment for the included trials (Fig. 7), SM-BOSS 2025 and SLEEVEPASS 2022, indicates generally low risk across most domains. Both studies demonstrated adequate random sequence generation and allocation concealment, suggesting a low risk of selection bias. Similarly, blinding of participants and personnel was considered low risk, indicating that performance bias is unlikely to have influenced the results (Fig. 8). However, some concerns were noted regarding outcome assessment and incomplete outcome data, which were classified as unclear. These findings suggest a potential for detection and attrition bias, mainly due to incomplete reporting or lack of clarity regarding the blinding of outcome assessors. Selective reporting was judged as low risk in both trials, and no additional sources of bias were identified. Overall, the global risk of bias for both SM-BOSS 2025 and SLEEVEPASS 2022 was rated as low. Thus, although the methodological quality is generally sound, caution is warranted when interpreting results related to outcome assessment and missing data. These limitations should be considered in the synthesis of evidence and in future research planning.

### Risk According to Robins I

**Fig. 9:**
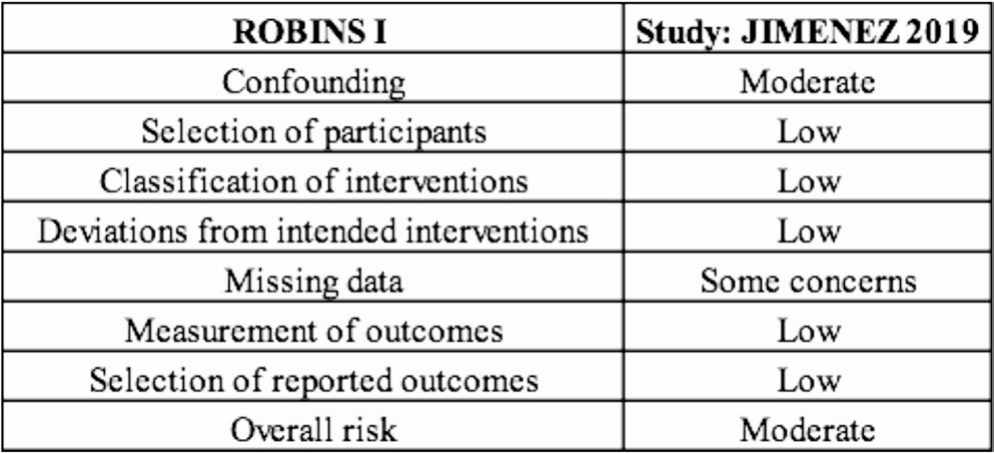
Risk of robins i

Jimenez et al. (2019) evaluated the long-term outcomes of vertical gastrectomy (SG) compared to Roux-en-Y gastric bypass (RYGB) during a 10-year follow-up period in a prospective non-randomized cohort. According to our assessment using the ROBINS-I system, the study.

presented a moderate risk of bias due to confounding factors, while other domains (including participant selection, classification of interventions, deviations from intended interventions, measurement of outcomes, and selection of reported outcomes) were assessed as low risk (Fig. 9). From this perspective, the study by Jimenez et al. contributes valuable long-term evidence on the comparative effectiveness of bariatric procedures, although its non-randomized design and potential confounding factors should be considered when interpreting the results. Thus, the overall moderate risk of bias highlights the need for cautious interpretation, especially regarding the reported differences in the remission of the comorbidities evaluated.

## Discussion

This long-term meta-analysis, based on randomized clinical trials and a prospective cohort with ≥ 10 years of follow-up, provides a comparative assessment between vertical gastrectomy (SG) and Roux-en-Y gastric bypass (RYGB). Thus, both procedures demonstrated sustained statistical efficacy in weight reduction and remission of metabolic comorbidities, with no statistically significant differences in the magnitude and durability of the effects.

In this sense, RYGB showed a statistically non-significant advantage regarding the average weight loss than the SG and did not show greater variability in weight results compared with the RYGB. On the other hand, SG shows a higher rate of surgical conversion, mainly to RYGB, because the conversion is relatively easy to do in case of weight loss failure or refractory gastroesophageal reflux [1, 6, 7]. Regarding remission of type 2 diabetes mellitus, dyslipidemia, and systemic arterial hypertension, the results were comparable between procedures, although some studies show a statistically non-significant tendency for RYGB to be more beneficial for systemic blood pressure due to greater neurohormonal modulation and changes in intestinal sodium absorption [8, 9, 2, 3, 10].

These data corroborate that both SG and RYGB promote a significant metabolic impact, consolidating both as viable therapies for obesity complicated by cardiometabolic comorbidities. Regarding the long-term safety profile revealed statistically non-significant differences between both techniques, however SG was associated with a slightly higher incidence of new or exacerbated gastroesophageal reflux [1, 6, 7]. It is also critical to acknowledge that this long-term analysis does not capture early major adverse events; the reputation that RYGB may carry a higher risk of initial mortality and emergency reoperation is a significant factor in patient choice that should not be omitted from the surgical consultation.

Clinically, patients with pre-existing or erosive reflux disease may benefit preferentially from RYGB, while SG may be indicated for individuals with lower surgical risk or preference for a restrictive procedure, provided they are monitored and evaluated for late complications [1, 2, 6]. Therefore, these findings reinforce the need for individualized surgical planning, considering baseline comorbidities, anatomy, surgical risk profile, and tolerance to anatomical changes.

 [2, 3, 10, 11].

Among the limitations of this analysis are the relatively small number of randomized trials with ≥ 10 years of follow-up and the inclusion of at least one observational study, which makes it difficult to interpret long-term results, especially in intention-to-treat versus per-protocol analyses. Future studies should prioritize standardized definitions of comorbidity remission, robust assessment of late complications, and stratified analysis by clinical subgroups [1, 6–8].

In summary, RYGB and SG has a similar metabolic profile and long-term weight loss with no statistically significant difference between both procedures. Thus, the choice of procedure should be individualized to optimize clinical and metabolic outcomes for patients [7–9, 12–14].

## Conclusion

The decadal outcomes of sleeve gastrectomy and Roux-en-Y gastric bypass reveal a level of parity that challenges historical procedural endorsements. Fidelity to the data indicates that ten years after surgery, there is no statistically or clinically significant difference in total weight loss or excess weight loss between the two techniques. Furthermore, the remission of major metabolic comorbidities, including type 2 diabetes and dyslipidemia, is comparable across both cohorts, suggesting that the initial physiological advantages of the bypass may equilibrate over the long term.

## Data Availability

No datasets were generated or analysed during the current study.
